# Exploring the Intersection Between Violence Against Women and Children from the Perspective of Parents Convicted of Child Homicide

**DOI:** 10.1007/s10896-018-9964-5

**Published:** 2018-04-12

**Authors:** Bianca Dekel, Naeemah Abrahams, Michelle Andipatin

**Affiliations:** 1 Present Address: Gender and Health Research Unit, The South African Medical Research Council, Francie van Zijl Drive, Parowvallei, Cape, PO Box 19070, Tygerberg, Cape Town, 7505 South Africa; 2 Psychology Department, The University of the Western Cape, Bellville, South Africa

**Keywords:** Violence against women and children, Child homicide, South Africa

## Abstract

Violence against women and violence against children are distinct research fields. Quantitative studies have demonstrated their intersection, but qualitative data provides an opportunity for a comprehensive understanding of this interface. Interviews with 22 parents/caregivers convicted of child homicide provided an opportunity to explore the context of violent experiences in their lives including their use of violence and their experiences of it in their intimate and parenting relationships. Using a feminist framework, we found that patriarchal family structures, gender and power dynamics contribute to the use of violence. Revenge child homicide was common with distinct gendered differences. This study calls for closer collaboration between the two fields to assist in developing prevention interventions to address and eradicate both forms of violence.

## Introduction

Violence against women and violence against children are public health concerns, with devastating consequences (Butchart and Mikton 2014). Global data for violence against women, from 141 studies in 81 countries found 30% of women, 15 years and over, experienced Intimate Partner Violence (IPV) (Devries et al. 2013). For children, Hillis et al. (2016) collected data from 112 studies in 96 countries, estimating that the number of children, 2–17 years, exposed to emotional, physical or sexual violence in 2015 exceeded one billion. It has been acknowledged that violence against women and children overlap in the same household, and thus, it is imperative to understand and address potential intersections for prevention work (Guedes et al. 2016). Relatively few studies have assessed this overlap (e.g. Appel and Holden 1998; Chan 2011; Rada 2014). A qualitative study conducted in Uganda (Namy et al. 2017) explored this intersection bearing in mind gender and power hierarchies within the nuclear family, typically defined as encompassing a father, mother and children. The authors found patriarchal family structures created an environment that normalized violence and thereby reinforced women and children’s subordination. Women’s rights activists and researchers have long noted that patriarchal systems shape social expectations in order to uphold male superiority over women (Boonzaier and de la Rey 2004).

## Context of Violence Against Women and Children in South Africa

South Africa is a country with a capacity for extraordinary violence, with an exceptionally high rate of violence against women and children. Burton et al. (2015) conducted a national South African study, collecting survey data from 9730 adolescents,15–17 years old. They found that one in five young people had experienced some form of sexual abuse, one in three experienced physical abuse, and one fifth reported experiencing child neglect at some point in their life. Additionally, a national child homicide study found 454 children under the age of five were killed in 2009. More than half, 53.2%, were neonates, 0–28 days old, and 74.4% were infants, under 1 year of age, giving a neonaticide rate of 19.6 per 100,000 live births and an infanticide rate of 28.4 per 100,000 live births, which are amongst the highest reported rates (Abrahams et al. 2016). Finally, the most extreme form of violence against women is also not a rare event in South Africa. IPV is the leading cause of death among female homicide victims, with 56% of female homicides being committed by an intimate male partner, translating into one woman being killed by a male partner every eight hours in 2009 (Abrahams et al. 2012).

## Theoretical Framework

Feminist theory, through its focus on gender and power, has been a valuable lens through which to understand the intersection of violence against women and children. A paucity of studies incorporate a feminist framework to understand why parents kill their children, making this an important contribution (Dawson 2018). Feminist theory emphasizes the notion of power and control and is an inclusive theory as it considers how systems of power and oppression interact, not only amongst men and women, but also amongst parents and children (Brown 2004). Although we use an umbrella term, i.e. feminist theory, we acknowledge that feminism is not a unitary theory. Instead, it encompasses a range of theories that incorporate those that espouse adaptation of a traditional positivistic scientific model to promote women’s interests to those that advocate for the radical separatist feminist position (Bunting and Campbell 1990). The aim is not to homogenize different strands of thinking into one convenient label (Morrissey 2003). Additionally, although the term ‘women’ is used, it is recognized that there are differences amongst women; however, this is not to say that there are no commonalities amongst women as well as differences. Thus, ‘women’ may no longer be thought of as a unitary category but it can still potentially be a unifying one (Jackson and Jones 1998). Such terms stand for the social construction of a particular set of people facing – albeit with large differences – a common reality based on a common oppression (Letherby 2003).

## Men’s Use of Violence

Dobash and Dobash (1981) emphasize that violence against women is an expression of male domination, a cultural phenomenon stemming from a history of sanctioned abuse and ownership of women. Although no longer legally sanctioned, feminist theory proposes that, the underlying culture of inequality persists through the expression of gender roles and social norms (Ritter et al. 2014). Therefore, this theory is grounded in the principle that violence against women is the result of male oppression of women within a patriarchal system, and takes into account how traditional ideas about marriage, the family and gender roles support male dominance. It is argued that patriarchal structures promote a hierarchy, with men in a superior position to women and children, and that it is within this that men’s power is created, and continued, in the family, where men’s dominance is demonstrated and reinforced, legitimizing violence as a form of control over “subordinate” family members (Boonzaier and de la Rey 2004). Indeed, patriarchal structures can create perceptions of ownership of the entire family, or of the children, leading to the dehumanized status of the subordinate family members.

In addition, it is purported that men, who hold these patriarchal stereotypes, tend to blame women and children for breaking expected dutiful, submissive behaviors, thereby validating violence as a legitimate form of social control (Namy et al. 2017). It is believed that abusive men experience feelings of power and control in choosing to use violence to solve conflict in relationships (Lebow 2012). When a husband beats his wife or child, feminist researchers, such as Brown (2004), maintain that these forms of behavior are interpreted as strategies for upholding the oppressive cultural status quo. Tied to this is that, in many societies, as in South Africa, men’s use of violence is largely considered normal (Morrell et al. 2012). There remains a tendency for men to be exonerated, as their violence is viewed as uncontrollable, a “natural” response to the stress associated with masculinity and as a result, there is a tolerance for men’s violence as an expression of anger, associated with notions of hegemonic masculinity (Namy et al. 2017). Researchers have also proposed that many South African men act violently to claim a dominant position over women and children. This may be due to their inability to control them through other means as they, for example, may experience social and economic marginalization, affecting their ability to live up to traditional standards. Simultaneously, these men are competitive about power, status, and honor. This combination of lacking the means to establish dominance and an unwillingness to accept a non-dominant position has been described as a root cause of violence against women and children (Lindegaard 2017).

Furthermore, Damant et al. (2008, 2009) remind us that women’s mothering is also targeted in men’s violence, highlighting the double level of intentionality whereby a violent act directed towards one individual, e.g. mother/child, is simultaneously intended to affect the other. Thus, maternal violence cannot be understood without situating it within the broader context of a patriarchal family structure, which disempowers women.

## Women’s Use of Violence

Feminist theory maintains that women’s violence stems from their victimization and oppressive experiences as women and mothers. It is within the home environment that mothers may endure trauma associated with witnessing a partner’s use of violence against their children. Mothers are also often abused in front of their children, which may trigger shame/embarrassment, compromising their role as mothers, as men’s violence affects all aspects of women’s lives, including their physical and mental health, which makes it difficult to perform the already strenuous work involved in mothering (Damant et al. 2009). It is within this oppressed, abusive, male dominated home environment, that women may displace their anger onto children. Women may violently express their powerlessness - or attempt to consolidate their power - over children, the most subordinate within the hierarchy (Namy et al. 2017). As such, children represent a source of both power and oppression for mothers as many women hold minimal power both outside and inside the home, relative to men, yet simultaneously are in positions where they are responsible for childcare.

However, Fawcett and Featherstone (2000) maintain that women should not always be constructed as passive and powerless victims, suggesting that the power dynamics are far more complex and dynamic, playing out differently in various contexts. Consequently, we acknowledge that there is a tendency amongst feminist researchers to remain within a relatively ‘safe’ and familiar realm by explaining women and men’s violence through emphasizing female victimhood/female passivity and male oppression/male power, instead of exploring the potential for agency (Morrissey 2003). Thus, we acknowledge that participants’ use of violence involved a degree of choice from within a range of possible choices and therefore, one could ask whether murdering a child could be interpreted as a form of agency, whether consciously or unconsciously, in the circumstances of the lives of these participants’. We agree with Allen (1987, p. 94) who warns that we should not follow their narratives, “into suppressing the recognition that these men/women can also – even at the very moment of their victimization and coercion – be conscious, intentional, responsible, and potentially dangerous and culpable”. Therefore, context enables and produces, but does not determine crime, and its consideration need not negate agency and responsibility (Morrissey 2003).

## Study Rationale

While the recent focus in the intersection between violence against women and children has generated meaningful contributions, research gaps remain. International literature suggests it is likely that maternal child homicide perpetrators have been victims of IPV (e.g. Friedman et al. 2005) and that paternal child homicide perpetrators have perpetrated abuse towards an intimate partner (e.g. Cavanagh et al. 2007). Qualitative research provides the opportunity to explore how parents, defined herein as biological, step and de facto parents, convicted of child homicide experience intersecting violence. Existing research largely originates from high-income settings where social and gender norms and structural hardships differ from low-income contexts (Guedes et al. 2016). Although many violence against women studies are approached from a feminist perspective, few of these studies have focused on the connection between violence against women and children. This paper seeks to address these gaps as, to the best of our knowledge, there is no South African qualitative study exploring the overlap between violence against women and children from the perspective of parents convicted of child homicide. Finally, a recent global systematic review aimed at describing child homicide perpetrators found that children face the highest risk of homicide by parents (Stöckl et al. 2017). In addition, since the South African national child homicide study found a parent was the perpetrator in the majority of cases (Abrahams et al. 2016); this study focuses on parents.

## Methods

### Sampling and Recruitment

Research ethics approval was obtained and interviews were conducted with 22 participants, refer to Table 1 for participant information, who were incarcerated for the death of a child. For convenience, interviews were conducted at five correctional centers in the Western Cape Province of South Africa. These centers were classed as both Medium and Maximum centers and were located in both urban and rural areas and housed a diverse group of men and women, as they house offenders convicted for crimes ranging from robbery to murder and with various lengths of sentencing. The study’s sample size could be viewed as a limitation as it may not appear representative of the population of parents convicted of killing their children in South Africa. In addition, these narratives stem only from those men and women who have been formally convicted, thus, the study has the limitation of being an offender based sample rather than a sample of all parents who have killed a child. However, Parker (2005) reminds us that qualitative research is not concerned with a large sample size or with the generalizability of findings, but rather with the richness of human narratives. Nevertheless, our sample encompasses men and women stemming from different racial categories, different ages, and educational levels. See Table 1.

**Table 1:** Sample characteristics

Name	Age	Race	Victim age	Victim’s manner of death	Relationship to victim	Highest educational level	Abused partner/was abused by partner	Number of interviews conducted
Michelle	23 yrs. old	Colored (i.e. mixed ancestry)	2 yrs. old	Fatal child abuse	Caregiver	Grade 6	Yes	2
Thandi	31 yrs. old	Black (i.e. African ancestry)	3 yrs. old	Fatal child abuse	Mother	Grade 10	No	1
Deidre	22 yrs. old	Colored	1 yr. and 2 mnths	Neglect	Mother	Grade 7	Yes	2
Latifa	36 yrs. old	Colored	5 yrs. old	Fatal child abuse	Stepmother	Grade 12/Matric	No	2
Winnie	20 yrs. old	Black	1 wk. old	Asphyxiation	Mother	Grade 4	No	1
Nicole	34 yrs. old	Colored	2 yrs. old	Fatal child abuse	Mother	Grade 9	No	2
Ryan	32 yrs. old	White (i.e. European ancestry)	2 yrs. old	Fatal child abuse	Caregiver	Grade 10	No	2
Adam	45 yrs. old	Colored	12 yrs. old	Strangulation	Father	Grade 10	Yes	3
Michael	53 yrs. old	White	Child 1: 16 yrs. old, Child 2: 5 yrs. old, Child 3: 21 mnths old	Firearm	Father	Grade 12/Matric	Yes	3
Zolu	33 yrs. old	Black	2 yrs. old	Asphyxiation	Father	Grade 11	Yes	2
Abigail	34 yrs. old	White	6 mnths old	Sharp injury	Stepmother	Grade 12/Matric	No	1
Lauren	28 yrs. old	Black	Newborn	Sharp injury	Mother	Grade 11	No	2
Zubeidah	34 yrs. old	Colored	2 yrs. old	Smothering	Mother	Grade 10	Yes	3
Christelle	36 yrs. old	Colored	6 mnths old	Smothering	Mother	Grade 12/Matric	Yes	3
Cayleigh	32 yrs. old	Colored	7 yrs. old	Smothering	Stepmother	Grade 10/Matric	Yes	3
Patricia	32 yrs. old	Black	1 yrs. old	Poisoning	Mother	Grade 11	No	2
Jennifer	29 yrs. old	White	3 mnths old	Fatal child abuse	Mother	Grade 9	Yes	3
Nelly	41 yrs. old	Black	9 yrs. old	Fire injury	Mother	Grade 7	No	2
James	36 yrs. old	Colored	5 yrs. old	Fatal child abuse	Stepfather	Grade 3	Yes	2
Jamaal	38 yrs. old	Colored	8 yrs. old	Strangulation	Father	Grade 10	No	3
Sipho	30 yrs. old	Black	2 yrs. old	Fatal child abuse	Father	Grade 8	Yes	3
Howard	33 yrs. old	Colored	2 yrs. old (twins)	Sharp injury	Stepfather	Grade 9	Yes	2

Recruitment began using purposive sampling through utilizing each correctional center’s psychology department. Correctional center psychologists identified men and women who were incarcerated for the death of a child in their care, and asked each person whether he/she would be willing to participate. If he/she agreed, a suitable date and time was arranged. Fourteen participants were identified using purposive sampling. Eight participants were identified using snowball sampling. Once interviews commenced, offenders voluntarily provided additional names of offenders known to them who met the study criteria and all participants agreed to participate.

### Data Collection

During interviews, the first author was present, as well as the participant and the relevant translator. To ensure confidentiality, members, i.e.wardens, did not sit inside the interview rooms. Participants had a choice as to their preferred language for the interviews. An Afrikaans and an isiXhosa translator accompanied the first author when needed. Each interview was recorded, translated and transcribed verbatim into English. A detailed explanation about the aim of the study, research procedures, risks and benefits, and rights of the participants was provided before written informed consent was sought. To maintain anonymity, pseudo names are used, for both participants and victims. Since this study was conducted in a correctional center, an incentive was not provided to those who agreed to participate. Psychological support for participants was an imperative part of the study and therefore, prior to each interview we asked each correctional center psychologist whether they would be willing to meet with offenders after the completion of interviews, if the offender felt this was needed. All correctional center psychologists agreed and five participants were referred. Psychological support was also arranged through institutional counselling services for the researcher who conducted the interviews.

Individual, semi-structured interviews were conducted, which involved open-ended questions to allow participants to freely express their thoughts. Each interview ranged between one to two hours. A scope of enquiry was developed and used to guide the interviews. The first interview explored the participants’ childhood and adolescent experiences, centering around their relationships with their parents. Examples of questions asked include, “Tell me about your childhood life” and “Tell me about your mother/father”. The first interview steered the second interview, which focused on their relationships with their spouses and children, their desire to be a parent as well as the factors surrounding the actual death of the child. Examples of questions asked include, “How did you meet your spouse?”. The third interview entailed follow up questions and provided participants with an opportunity to elaborate or provide further detail.

### Data Analysis

Atlas T.I 8.0 was used to assist in data management, which was performed according to the grounded theory principles of open, axial and selective coding (Corbin et al. 2014). During open coding, the first author examined the texts for salient categories of information supported by the data. The aim was to have concepts emerge naturally without forcing them into predefined categories. This stage also encompassed comparing incidents with other events to search for similarity and differences, which were then, grouped together and assigned codes. Categories were divided into sub-categories, which was crucial as there were 108 codes created. As analysis progressed, these codes were refined with the assistance of the second author concluding the initial coding stage with 54 codes. Together, during axial coding a single category was identified as the central phenomenon of interest, positioned as a main feature, and the data was returned to with the aim of finding additional information to understand the categories relating to this phenomenon. Further, in this phase of analysis, the relationships between the categories and sub-categories are highlighted. The final coding stage integrated all work done in the first two phases: entailing the identification of a core category/central phenomenon, i.e. most extensively discussed by participants, around which, other categories were related and a storyline was constructed. To illustrate this storyline, discriminate sampling was used, which refers to selecting certain participant quotes, which are able to maximize opportunities for verifying the storyline. After conceptualizing the storyline, it needed to be validated, which was performed by searching for relevant literature pertaining to the categories in order to validate its meaning; member checking with participants was conducted to iron out ideas and reach consensus; and discussing the storyline with the co-authors. Finally, core categories were then organized into the research paper.

This study aimed to capture the individual’s unique story/narrative. The intention was not to obtain the “truth” but rather to capture the participants’ subjective experiences. We acknowledge that the issue of the “truth” of offenders’ stories is particularly thorny and that this may be complicated by an inclination to cast oneself in a positive light. Thus, the aim was to accept the offenders’ stories as their reality as it is their subjective perspective of their reality (Sandberg 2010). Having said that, one could argue that a potential limitation is the absence of differing perspectives of the crimes, e.g. partners, grandparents. However, this was beyond the scope of the study.

### Findings

This paper is about the intimate relationships, between the participants and their romantic partners. Their relationships were marked by hardship and abuse and as a result, speaking about their relationships was emotionally difficult. Violence in their intimate relationships was often not their first violent encounters with many speaking about physical and emotional abuse by both fathers and mothers during childhood and adolescence. Throughout the interviews, most participants reported failure by a parent to protect them from abuse perpetrated by the other parent/stepparent, abandonment and rejection by either a mother or father or both parents. As a result, some participants were raised by grandparents, mainly grandmothers, and tended to move around a lot, resulting in unstable childhoods for many. They consequently struggled to form healthy bonds with their own children and many experienced difficulty transitioning into a parental role. For some, this was followed by wanting to be ‘different’ fathers and mothers as compared to their parents.

### Intersecting Violence Against Women and Children Within South African Families

#### The Men’s Narratives of Abuse

The intimate relationships of the men and women incarcerated for killing a child were rife with abuse. Six of the eight men admitted to abusing female partners. It appeared as though these men responded to their partners and children in similar, abusive manners when they believed that their partners and children acted in a way that served to question their authority; i.e. when the women and/or children did not act in accordance with expected submissive behaviors. To these men, who were raised within patriarchal cultures, violence was seen as a legitimate form of control.

For example, Adam aged 45 years old, and whose father was murdered when he was younger, was convicted of the murder of his 12 year old daughter,. Adam explained that when his wife and mother of his children upset him, he would resort to violence: “If I get cross with her, I smack her. If we quarrel, I smack her”. Likewise, if his daughter did not abide by his rules, he resorted to violence. Shortly before Adam murdered his daughter, he discovered that she had not abided by his rules and that she had gone to party where alcohol was served and as a result: “I smacked her and her head hit against the door and she was screaming”.

Similarly, James, aged 36 years old, who was also abused as a child by his alcoholic father, was convicted of the murder of his stepson, aged five years old, whom he physically beat to death. James’s mother passed away when he was four years old and he battled to cope with this loss, as he seemed to have attachment issues: fear of being abandoned by a partner and difficulty with emotional intimacy in relationships. He reported turning to violence if any of the women he was having a sexual relationship with, went against his rules by informing outsiders that they were in a relationship: “I will hurt them if they tell people we are like boyfriend and girlfriend”.

A dominant feature within the men’s narratives was that of control and punishment of their spouses’ perceived wrongdoings, which ultimately ended in the murder of a child to enact revenge, i.e.; they punished their partners through killing a child in a deliberate attempt to make their female partners suffer, emotionally. These parents may be viewed as displaying excessive or disordered control, which was expressed through taking a child’s life (Sidebotham 2013). Michael, aged 53 years old, who killed his three children, explained his controlling behavior within his marriage: “I started to write down and check the kilometers on our car every day…. If there was extra mileage then it means she is driving somewhere else, other than just to work and back… On the day of the murders, I checked the additional kilometers again … and then I killed my children”. His quote alludes to his wife’s perceived unfaithfulness and he proceeds to explain that upon learning that she was unfaithful: “I felt disappointed. I felt disgusted…I kept the anger inside and that evening, I exploded”. Similarly, in referring to his then wife and mother of the child he killed, Jamaal, who also experienced abandonment and rejection by both his parents, explained how he felt upon discovering that she had been unfaithful: “It was almost like a volcano inside of me that wanted to erupt…After that my anger just grew more and more inside of me. I was angry you see, just to think about it… Never in the deepest chamber of my mind did I think this is gonna occur… Love betrayed me”. This incident occurred shortly before he killed their eight year old son.

Zolu, who has never met his father, also killed his son to enact revenge against his then girlfriend and mother of his child, Nicole, who he admitted to beating with “a belt”. He killed his two year old son shortly after she had laid a charge of rape against him. He disagreed with her and told us: “there was no force” and that it was consensual. Whether his account is a true or misleading representation is unknown. However, the sadistic manner in which he dealt with it is revealing. In a fit of rage, Zolu buried his son alive: “I was angry inside… My aim to do that, I wanted to make her heart sore… I wanted her to feel the pain…. I didn’t want to kill her because then she won’t suffer and she must suffer…I thought when she die, she won’t feel any pain. I wanted Nicole to feel a pain, that’s why I did what I did. She love that child…You reap what you sow. You must reap, you don’t just do things without like a consequence for what you doing”.

Sipho, who was raised by his grandmother, was the only male participant who was also convicted of an attempted murder of a female partner. At the age of 24, Sipho tried to kill his then girlfriend, but accidently killed his son instead. He physically beat his two year old son to death and attempted to kill his girlfriend. In discussions around his childhood, Sipho reported that his mother was absent since birth and according to him, he had an emotional void, which he was unable to fill, until he met his girlfriend, who he felt was the only woman able to fill this emptiness, making her a prized possession. As with Michael and Jamaal, on the day that Sipho killed his son and attempted to kill his girlfriend, he had become suspicious of her faithfulness and consequently: “I started hitting her. I didn’t realize she will cover her face or her chest with my son… She held his head in front of her face and her chest…I was angry”.

#### The Women’s Narratives of Abuse

Half of the female participants reported experiencing IPV. Many of these women explained that their male partners controlled and isolated them and that this isolation contributed towards the killing of their children as the burden of household and childcare, coupled with an absence of a support structure while enduring IPV, was too strenuous for them. For instance, Christelle, 36 years old and previously unemployed, explained that: “I ended up with no friends when I was with him… he just cut me off everybody and I ended up having no support” and that “all of this became too much for me to handle”.

Many of these women had been victims of abuse from young ages. For example, Zubeidah, who was convicted of the murder of her two year old son at the age of 30, mentioned that she experienced abuse herself as a child by her parents and that this abuse: “stopped when I got married… cause I couldn’t take it anymore. That was my scapegoat. To get pregnant, to get out of the house… But I was ever so sorry that I got married”. Once she met her husband, he also “abused me physically and emotionally”. She stated that because of this abuse: “I couldn’t look after my son properly”. On the day, she killed her son: “I was so angry and I had a flashback of everything that I went through with my husband; how he abused me, how he neglected Taahir” and because of all of these factors believed that “he was much safer in Heaven… I killed him so that he can no longer suffer here on earth”.

Another participant was also abused as a child by her mother and as an adult, by her fiancé and father of her baby: “he started to abuse me. There was days that went by that he hitted me from the morning until the next morning”. At the age of 21, Jennifer was found guilty of fatal child abuse of her six month old daughter, where again, the couple were intending to abuse each other and accidently killed their daughter: “We started to fight over the child, with me forgetting that she’s on the bed. I started to hit without realizing I’m hitting her. So I hit and he started to hit and as he hit I lost control. I hit back without realizing it was her”.

Similar to the male participants’ narratives, one woman, who was the only participant to experience sexual abuse perpetrated by a parent, resorted to killing a child to enact revenge against her unfaithful and abusive male partner. At the age of 23, Cayleigh murdered her seven year old stepdaughter, her husband’s daughter: “I was angry… I’m sitting in prison, not because I wanted to kill Bernadette, but because of hate. I just wanted to get back at him for hurting me. To make him feel pain, because he was making me feel pain inside”.

Christelle was the only woman who was convicted of the double murder of both her six month old son and of her abusive boyfriend. She also attempted suicide after committing the murders. She explained that this was not an attempt to enact revenge, but was rather a means to escape her abusive relationship: “I was thinking everything was too much and that was the easiest way out”.

Many women experienced a lack of financial and physical support, which contributed to the homicide. Overall, except where addiction comes into play, the women presented themselves as hardworking and financially supporting their children to the best of their ability, given their limited education, which often locked them in poverty. However, some women were financially dependent on their male partners, which meant they had fewer options for leaving. For example, Christelle stated that: “I couldn’t leave him because I was like, so dependent on him because I needed money for the baby and for me”. In these situations, the men were able to use their position of economic power to exert control over their women and abused their power, perpetrating financial abuse, by purposely giving their partners a limited amount of money for household/child expenditures. She explained: “He just like paid R100 when he feels like it and that money will help me out for two, three days. But then I was still stressed about where am I going to get again after that. He did that on purpose to make me stress, because he had enough money to give me, he had a good paying job”.

Many of the women’s narratives depicted misplaced anger. Some mothers directed their frustrations stemming from a lack of support from partners or feeling as though the abuse worsened after their children were born, onto their children. For example, some women maintained that their financial strain worsened with the birth of their children, as Christelle stated: “I saw him (son) as, like, this problem in my life, because although there were already financial problems before him, when he arrived the problems just added up more, so at the back of my mind, I blamed him. Because if he wasn’t there, I wouldn’t have struggled going through all that”. Likewise, Zubeidah mentioned that: “After he was born, then the abuse got worse” and likewise, Christelle said: “When he arrived… like the abuse got worse… and I blamed him for the added abuse”. Both women reiterated that these strains were too overwhelming, which may have largely contributed to the homicides.

## Discussion

To the best of our knowledge, this is the first South African qualitative study exploring the intersection between violence against women and children from the perspective of parents convicted of child homicide.

### Revenge Homicides

This study has shown that both parents killed children in attempts to enact revenge against a partner. Revenge child homicides are explained as anger directed at the, ex or current, spouse, without the motive to kill the spouse; instead, the motive to kill is directed towards the child, as the perpetrator knows this will cause long-term emotional harm to the spouse, which is the end goal (Resnick 1969). In essence, these individuals desire to hurt their current/ex-partner, as they themselves are emotionally hurting and thus, want their spouse, the person they believe inflicted this pain on them, to also feel emotional pain, and believe that killing the child will cause this harm and so they act in a way consistent with these mental states. The murder stems from anger towards a spouse, which is misdirected and aimed instead towards the child (Carruthers 2016) and it was evident in the participants’ narratives that these murders were often motivated by anger. The anger towards the spouse serves as a trigger, igniting far greater and deeper anger, which often stems from their experiences of childhood abuse. However, we acknowledge that being angry with one’s partner does not necessarily lead to child homicide.

Gollwitzer et al. (2011) view revenge as a form of communication between the perceived “victim”, the perpetrator, and the offender, (their spouses. “Victims” seek to deliver a message to their offenders: you will be punished for what you did; for example, in this study, for either allegedly being unfaithful or for opening a rape case. The desire to retaliate is a universal phenomenon and is related to our moral intuitions and our notions of justice, e.g.: “She has to suffer… You reap what you sow. You must reap, you don’t just do things without like a consequence for what you doing”. In South Africa, there is also the normalization of violence as a conflict resolution response (Nagy 2004). Michael, Jamaal, Sipho, and Zolu were all faced with the same situation: discovering, whether true or not, that their partners had in some way betrayed them. According to feminist theory, for these men to regain a sense of manliness, the perceived betrayal had to be punished. Not only was the murder a deliberate and final act to regain control, but also a way in which to punish their spouse. These men externalized blame and attributed the breakdown of their relationships and the actual killing as being triggered by their partner’s behavior. They idealized their once ‘perfect’ women who were now flawed due to their perceived behavior, which was not fitting. This type of behavior reflects an intense vulnerability and the mistrust often develops due to their own insecurities. Their fragile sense of self allowed these men to polarize their partners into all good or all bad (Mathews et al. 2014).

### Dehumanization of the Victim

Our findings revealed that there were variations in the manner in which these men reacted. Michael, Jamaal, and Zolu made no attempts to physically harm their female partners and in contrast, Sipho intended to physically injure his girlfriend and instead, accidently killed his son. However, all four of these men killed their own, biological children. In rational parenting, the desire to protect one’s child surpasses everything else. Murdering one’s child violates societal norms of morality and of the parenting role (Adinkrah 2001). How were these men able to treat their own children, the paradigmatic in-group members, as outsiders, an inferior other, who is less than human and so killable? Why did these men, unlike Sipho, not treat the child’s mother as killable? Carruthers (2016) proposes that these men do not view the child as a human being, worthy of protection and having a future, instead, they view the child not as their child, but as a thing, a mere object, which erodes part of the problem of explaining this specific type of killing. If these parents view the child as a mere possession, they will not assign themselves the role of caregiver and thus, the power of the caregiving norm does not need to be overcome in order to murder the child. It is, thus, more understandable how the aspects identified above could lead to homicide. Therefore, it may be that in the murderers mind, he/she is not killing a person at all, but is instead destroying something of monumental emotional significance to the target. Revenge killers fail to represent the personhood of the murdered child and instead perceive the child as an extension of the ex-partner or as a thing. This failure also explains why the motivation to protect one’s child is not prompted and does not prevent the motivation to kill. As the child is perceived as an object, it has no welfare for the murderer to protect. This also explains why the spouse themselves were not targeted and explains how these men and women were able to understand that murdering the child will emotionally hurt the other parent. Alternatively, these men could also have shifted their way of viewing the child, from an in-group member to an out-group member, as a result of the betrayal (Carruthers 2016).

Only one woman, Cayleigh, committed revenge child homicide in response to her male partner’s infidelity and abuse. A difference however, between her and the men’s narratives, is that these men killed a biological child, whereas she killed her stepchild. Thus, it is not as complex as to why her stepdaughter was killed; the child of an unfaithful husband would easily be treated as an out-group member. In addition, Calyeigh killed her stepdaughter to enact revenge for the prolonged abuse she had endured: again, a key difference between her narrative versus the men’s.

Christelle was the only woman convicted of killing a male partner. She killed her boyfriend and son and attempted suicide thereafter, which was in response to prolonged abuse she had endured. Her case could rather be classified as familicide, as the goal was to annihilate the entire nuclear family (Liem and Reichelmann 2013). The gendered difference here is that none of the men in this study killed in response to being victims of IPV. It has been demonstrated that some women may commit murder in response, or in self-defense, to enduring abuse (Atkins et al. 1999; Miller 2005; Peterson 1999; Smith et al. 1998). According to feminist theory, battered women who kill their abusers hold an anomalous position within Western heteropatriachal cultures. Their murders erode dominant discourses of traditional femininity as they challenge motherhood as a site of violence rather than perpetuating the myth of nurture and care. In addition, these women are viewed as both victims and perpetrators, as culpable yet blameless, inhabiting a paradox, where extreme victimization ultimately leads to lethal retaliation (Morrissey 2003).

Sipho was the only male participant who attempted to murder his female partner, which was an attempt at femicide, the most extreme form of violence against women. He displayed an exaggerated version of a dominant ideal of masculinity, by using violence. His behavior was a last attempt to take back control in a context where gendered relationships legitimize men’s use of violence to assert power. Lau (2009) posits that abusive men tend to adopt rigid, stereotyped views on what is expected from a woman, for example, that she must be faithful and that she must obey her husband. Men often resort to violence when their partners are seen to violate these gender norms and thus, the violence was an attempt to punish her for her perceived betrayal (Mathews et al. 2014).

### Women’s’ Use of Violence

Jennifer’s narrative resembles that of Christelle and Sipho’s: As with Sipho, Jennifer’s daughter died as a result of being an innocent bystander (Guileyardo et al. 1999). However, as with Christelle, Jennifer’s actions were in response to enduring prolonged abuse. According to feminist theory, women’s choices and actions need to be understood within the context of oppression and in this case, within an extremely abusive context. Nevertheless, these women acted as active agents who made clear choices to perpetrate violence. Women may occupy a range of subject positions and may be victims in relation to their partners, while holding a position of power in relation to their children (Featherstone 1996). Therefore, their accounts present a discourse of shifting positions between powerlessness and agency (Hydén 2005).

Including women’s use of violence against children provides a valuable opportunity to contextualize maternal violence within a feminist understanding of power and family hierarchies. This study showed that women often displaced their anger, thereby reacting violently towards their children after experiencing abuse from male partners and/or a lack of support from partners. Noteworthy is that none of the men killed for these reasons. The women placed blame onto their children, instead of aiming the blame towards their partner showing similar dehumanization of the child as in cases of revenge homicide. An understanding of this type of behavior cannot be separated from women’s systemic oppression within the family. Within this constricted environment, women may violently express their powerlessness, or try to consolidate their own power, over children, who are viewed as subordinate within the hierarchy. They may incorrectly take their feelings of frustrations out on their children (Damant et al. 2008). Conversely, the mother’s behavior may be interpreted as a distorted perception of protection from a harsh environment (Sidebotham 2013). Regardless, maternal violence needs to be situated within the broader context of the patriarchal family, as opposed to being understood as a specific incident or individual pathology (Namy et al. 2017).

It is vital to bear in mind that both men/fathers and women/mothers can perform a range of constructed subjectivities, which are shifting and sometimes conflicting. For example, as demonstrated in this study, mothers do not always perform the, stereotyped traditional gender role discourse, role of carer and nurturer and fathers do not always perform the role of protector. Thus, the interests of both fathers/mothers and children do not always coincide. This is not to say that women will not adopt the role of nurturer and that fathers will not protect their children, but there is a problem in assuming that they will always adopt these subject positions. It is crucial to recognize the complex power relations between women/mothers and children and between mothers and fathers, and the ambivalence that parents may feel toward their children, and therefore the possibility that parents will be violent toward their children (Damant et al. 2008).

We cannot ignore the impact that the participants’ childhood and adolescent experiences of parental abuse and neglect had on their perpetration of violence against their own children. It was illustrated in a previous paper (Dekel et al. 2018), that the abuse and absence of their own parents were remarkable and that for many, the psychological impact of an absent or abusive parent was lasting, leaving them emotionally vulnerable. As a result, many participants were unable to form bonds with their own children and struggled to assume a paternal or maternal role. Traumatic childhood experiences have a profound impact on future parent-child relationships and play a huge role in later adult violent behavior. It is therefore, imperative to reduce children’s emotional vulnerabilities by engaging in strategies to strengthen current parenting practices to promote the development of less violent parent-child relationships.

### Recommendations and Conclusion

Preventing violence against women and children requires a commitment to developing a holistic understanding of women’s experiences of mothering in the context of IPV as well as understanding men’s use of violence (Lapierre 2008). A few international feminist scholars have considered these issues (Damant et al. 2009; Radford and Hester 2006; Lapierre 2008, 2010). However, research of this nature is needed in South Africa, specifically focusing on mother’s fatal abuse of children. This issue needs to be addressed to ensure that the complex power and gender dynamics within families will not be evacuated from the discussion in this area, as there is a need to intervene on both problems. Such work calls for a multi-levelled and multi-dimensional conceptualization of power, which considers gender inequalities. Researchers and policy-makers need to pay more attention to this issue, particularly from women’s own perspectives (Damant et al. 2009).

Furthermore, this study illustrated that women’s abuse of their children can largely be seen as a consequence of their own adverse experiences within a romantic relationship. In this regard, policy-makers and practitioners working with abusive mothers should be attentive to the circumstances in which these women perform their mothering, given that they may also be victimized by their partners. Thus, parenting intervention strategies need to be cognizant of this in order to be effective.

Although women’s behaviors cannot be removed from the context in which they occur, these women still have agency and their position as adults/mothers enables them to exercise power over their children and thus, their violence can be seen as an extension, or abuse, of this power. Noteworthy is that being victimized does not remove all responsibility, but we cannot ignore that it places these actions in a particular context. Further qualitative research is needed to understand the differences between those abused mothers who do and do not kill their children. It is also necessary to explore the positive strategies women adopt in these circumstances. Radford and Hester (2006, p. 145) suggest that, “the efforts that women living with violent partners may make to resist the violence and continue parenting on a daily basis are not adequately considered in the research literature”. Future research needs to adopt an analysis that centralizes issues of gender and power, and locates the problem clearly in men’s violence against women. However, this should not result in a one dimensional view of women as victims and should not remove focus away from the fact that they may hold multiple identities and live in different conditions, namely in relation to their mothering (Lapierre 2008). Overall, this study highlights the importance of developing a deeper understanding of the difficulties abused women face in relation to their mothering, which are at odds with the high expectations placed on women as mothers. As seen in this study, childcare was predominantly the responsibility of women. Men are to be encouraged to support their partners and active fathers are to be acknowledged.

Revenge child homicides are typically difficult to prevent, as there is usually little warning (Friedman and Resnick 2007). However, this study has shown that there is a tendency for IPV to precede revenge child homicides, committed by either a father or mother. We recommend that service providers such as South African Police Service members be educated around this intersection.

Tied to this, our findings highlight the need for service providers from all sectors to be prepared to recognize and respond to multiple forms of violence within families (Guedes et al. 2016). There are limited empirically validated violence against children screening tools that can be used within the South African context. A recent study confirmed that there is a need for safety and risk assessment tools pertaining to child protection in South Africa (Spies et al. 2017). There seems to be a general consensus within South Africa, for a variety of reasons, that the child protection system is failing our children (Jamieson et al. 2017). Jamieson et al. (2017) conducted research in an attempt to gauge an understanding of the functioning of the child protection system in South Africa. Some of their findings included that physical abuse is not taken seriously and rarely referred to the police by social services, allowing the abuse to continue and worsen, leading to a number of cases of fatal abuse. Further, the lack of therapeutic services risks increasing trauma, poor record keeping prohibits evidence based planning, poor case management and inadequate supervision lead to child being lost in the system, and children suffer because professionals are not working together. South Africa has a long way to go in ensuring children are kept safe.

In line with this, we support home and community based parenting programs, which have shown promise within both low, middle and high income countries, for reducing child abuse and which, may provide opportunities to address additional forms of family violence, such as violence against women (e.g. Bair-Merritt et al. 2010; Knerr et al. 2011). Evidence is emerging across the globe and in settings such as South Africa, that parenting programs have many positive effects on various public health problems beyond mere child abuse prevention (Skeen and Tomlinson 2013). Recently, a process evaluation of a parenting program to reduce the risk of child maltreatment amongst low-income families in South Africa was conducted. The findings indicated that parenting programs derived from evidence-based principles are feasible in South Africa when situated within a culturally relevant context (Lachman et al. 2018).

In conclusion, this study shows that violence against women and children intersect in a number of ways, and can no longer be understood as separate issues. The prevention of both forms of violence would benefit from a meaningful integrated approach. A comprehensive approach to working with families is needed to promote positive parenting practices. There is a particular need for interventions to focus on addressing gender inequality, the normalization of violence within the home, the patriarchal family structure, and transforming men’s power over women and children, in addressing violence against women and children (Fulu et al. 2017). The normative acceptance of men’s use of violence emphasizes that there is still much progress to be made. Finally, an exploration into the dynamics within non-violent families could complement the current analysis, revealing ways in which families defy existing norms and adopt more equitable relationships within the home (Namy et al. 2017).
